# The Need for Uniform Labeling Regulations for Monosodium Glutamate to Address Health Risks and Consumer Protection

**DOI:** 10.7759/cureus.90811

**Published:** 2025-08-23

**Authors:** Akhtar Purvez, Michael C Valentine

**Affiliations:** 1 Lifestyle Medicine, Lincoln Memorial University, Harrogate, USA; 2 Cardiovascular Medicine, University of Virginia, Charlottesville, USA

**Keywords:** atrial fib, food allergy quality of life, food intolerance, food science, monosodium glutamate (msg)

## Abstract

Monosodium glutamate (MSG) is a flavor enhancer commonly added to processed foods. Despite its GRAS (Generally Recognized as Safe) status by the FDA, accumulating clinical evidence, in the form of multiple case reports, links MSG to serious health risks in susceptible individuals, particularly those prone to atrial fibrillation (AF). Biological mechanisms, including glutamate receptor activity and oxidative stress, are believed to be the cause of this adverse effect. Moreover, inconsistent labeling practices disguise MSG under multiple names, making it difficult for sensitive consumers to avoid. The European Union, Australia, and Japan enforce stricter MSG labeling requirements than the United States to safeguard the susceptible population. This paper makes the case for uniform labeling regulations to promote transparency and protect at-risk populations from inadvertent exposure to MSG, advocating for standardized ingredient disclosure policies modeled on international practices.

## Editorial

Introduction

Monosodium glutamate (MSG) is widely used to enhance the umami flavor in various foods. While the FDA categorizes MSG as generally recognized as safe (GRAS), emerging evidence challenges this designation for specific vulnerable populations. While a causal relationship has not yet been established due to a lack of relevant studies, accumulating evidence in the form of case reports has correlated MSG with the triggering of atrial fibrillation (AF), a potentially life-threatening cardiac arrhythmia. The burden of AF is significant, contributing to increased risks of stroke, hospitalization, and mortality. For patients with MSG sensitivity, consumption of even small amounts may result in serious cardiovascular outcomes, warranting clear consumer protection strategies, beginning with standardized labeling. Researchers have linked MSG to adverse reactions like palpitations, flushing, and AF, as well as other adverse effects, especially in vulnerable populations [1-8]. An extensive review of the effects of MSG looked into its possible effects on human health [9], and another raised the question of its consumption as a threat to public health [10].Health risks of MSG and mechanistic insights

Multiple case reports indicate that MSG can induce AF in sensitive individuals [1-4,8]. Glutamate receptors, found in both nerve and heart tissues, might cause these effects by increasing activity and instability in the heart's electrical system. Additionally, MSG-induced oxidative stress and inflammatory cytokines, such as IL-6 and TNF-α, may contribute to atrial remodeling and the formation of an arrhythmogenic substrate [11]. These findings provide biological plausibility for MSG-induced cardiac effects. Scientific literature has described glutamate receptor activity in cardiac tissues and the role of oxidative stress in MSG toxicity [12].

Labeling challenges and regulatory gaps

A significant issue for consumers trying to avoid MSG is the lack of uniform labeling. It is frequently listed under other alternative and benign-sounding names (Table 1).

**Table 1:** Substances containing or releasing glutamates MSG: monosodium glutamate

Substances	
Monosodium salt	A sodium-based flavor enhancer with effects similar to MSG
Autolyzed yeast extract	Processed yeast product containing free glutamates, commonly used as flavoring
Hydrolyzed vegetable protein (corn)	Protein hydrolysate releases amino acids, including glutamates
Hydrolyzed corn gluten	Hydrolyzed corn protein acts as a flavor enhancer
Sodium caseinate (hydrolyzed protein)	Milk-derived protein stabilizer; contains free glutamates
Yeast extract	Rich in glutamates; used in soups, sauces, and processed foods
Textured protein (may contain MSG)	Plant protein (meat substitutes); may include added MSG
Calcium caseinate (hydrolyzed protein)	Milk protein derivative used in processed/nutritional foods
E621 (flavor enhancer)	International food additive code for MSG
Torula yeast	Yeast-based flavoring, naturally high in glutamates

This labeling ambiguity complicates the avoidance of MSG for individuals with sensitivities or a history of arrhythmia [3,4]. Misleading practices, such as "no added MSG" claims that still include free glutamate sources, further exacerbate consumer confusion, as highlighted in recent litigation against food manufacturers [13]. Although dismissed, the legal challenges underscore public concern and demand for clarity in food labeling practices. Furthermore, the integrity of the GRAS designation process itself has been called into question. Industry-connected experts approve many food additives as GRAS without adequate oversight, raising questions about transparency and public safety [14]. A recent report suggests that the FDA has fallen behind in regulating harmful food additives, advocating for reform of the GRAS loophole to restore GRAS to its original intent as a narrow exception for common ingredients with proven safety records [14,15].

The case for uniform and transparent labeling

We urgently need uniform labeling regulations to ensure public health safety. Countries like those in the EU require clear disclosure of flavor enhancers, including MSG. Regulation (EU) No 1169/2011-notably Article 9, which requires that additives like MSG be listed by both their category and specific name or E-number (MSG, E621) even when present within compound ingredients, and Article 7, which mandates that food information must not be misleading [16]. Australia and Japan also enforce strict labeling requirements. By contrast, the U.S. permits ambiguous ingredient designations that may obscure the presence of MSG. Aligning U.S. labeling laws with international norms would foster transparency, allowing consumers, especially those with cardiac sensitivities, to make informed dietary choices [5]. As with allergens like gluten and peanuts, MSG should be uniformly labeled to enable risk-aware consumption and promote patient safety.

Recommendations and public health implications

We recommend that the FDA and allied regulatory bodies implement the following:

(1) Mandate a standardized label for MSG and all glutamate-containing additives. (2) Prohibit the use of misleading claims, such as 'No Added MSG,' when free glutamates are present. (3) Incorporate MSG sensitivity training into physician education and nutritional counseling guidelines. Current literature shows concerning gaps-physicians may be unfamiliar with MSG sensitivity and its presentations, risking under-recognition when patients report relevant symptoms [17]. (4) Collaborate with industry stakeholders to phase in transparent ingredient disclosures.

Such reforms would not only protect vulnerable populations from adverse events like AF but also build public trust in the food labeling system. Transparent ingredient listing has been shown to improve outcomes in patients with food sensitivities [5,10]. This approach has global precedent, as seen in European regulations [16].

Conclusions

Given the emerging case and mechanistic evidence linking MSG to cardiac arrhythmias in sensitive individuals and the lack of consistent labeling, uniform MSG labeling should be prioritized as a public health intervention. Clear, standardized labeling would empower consumers, aid physicians in dietary counseling, and align the U.S. with global best practices. While adopting these practices for MSG labeling is feasible, we recognize potential industry concerns such as reformulation costs and consumer perception. These can be addressed through phased implementation and regulator-industry collaboration, as successfully demonstrated in the EU and Australia. Regulatory action on this issue can significantly reduce preventable health risks and reinforce the principle of informed nutritional choice. Policy reform must therefore consider scientific, regulatory, and clinical evidence supporting MSG’s potential harm [3,7,14,15].
